# Targeting SIRT1: A Potential Strategy for Combating Severe COVID‐19

**DOI:** 10.1155/bmri/9507417

**Published:** 2025-12-17

**Authors:** Hajar Shokri-Afra, Fatemeh Saber Jeyvan, Zeinab Barartabar, Parisa Khanicheragh, Elham Yousefi Abdolmaleki, Davod Ilbeigi, Hadis Musavi, Yalda Malekzadegan

**Affiliations:** ^1^ Gut and Liver Research Center, Non-Communicable Diseases Institute, Mazandaran University of Medical Sciences, Sari, Iran, mazums.ac.ir; ^2^ Department of Biology, School of Sciences, University of Guilan, Rasht, Iran, guilan.ac.ir; ^3^ Cellular and Molecular Biology Research Center, Health Research Institute, Babol University of Medical Sciences, Babol, Iran, mubabol.ac.ir; ^4^ Department of Clinical Biochemistry and Laboratory Medicine, School of Medicine, Tabriz University of Medical Sciences, Tabriz, Iran, tbzmed.ac.ir; ^5^ Department of Clinical Biochemistry, School of Medicine, Torbat Heydariyeh University of Medical Sciences, Torbat Heydariyeh, Iran, thums.ac.ir; ^6^ Health Sciences Research Center, Torbat Heydariyeh University of Medical Sciences, Torbat Heydariyeh, Iran, thums.ac.ir; ^7^ Department of Clinical Biochemistry and Medical Genetics, Faculty of Medicine, Mazandaran University of Medical Sciences, Sari, Iran, mazums.ac.ir; ^8^ Department of Microbiology and Parasitology, School of Medicine, Bushehr University of Medical Sciences, Bushehr, Iran, bpums.ac.ir

**Keywords:** COVID-19, cytokine storm, inflammation, SARS-CoV-2, signaling pathways, sirtuin 1

## Abstract

Sirtuin 1 (SIRT1) is a crucial regulator of cellular processes, including inflammation, metabolism, and stress responses, playing a significant role in the body′s defense mechanisms. During SARS‐CoV‐2 infection, SIRT1 plays a crucial role in modulating the immune response. This protein helps to enhance the antiviral response through deacetylating key transcription factors and regulating proinflammatory cytokines, thereby reducing the cytokine storm (an overwhelming immune response) associated with severe COVID‐19 cases. SIRT1 influences the expression of angiotensin‐converting enzyme 2 (ACE2), the primary receptor for SARS‐CoV‐2, thereby potentially mitigating viral entry and replication. Natural activators of SIRT1, such as resveratrol, have been shown to enhance its activity, offering promising avenues for therapeutic interventions aimed at bolstering the immune response during COVID‐19. Understanding the multifaceted role of SIRT1 in human defense mechanisms against SARS‐CoV‐2 could pave the way for innovative strategies to manage COVID‐19 and similar viral infections, emphasizing the importance of SIRT1 as a potential target for future therapeutic approaches.

## 1. Introduction

In December 2019, an unexpected epidemic caused by a novel coronavirus, SARS‐CoV‐2 (COVID‐19), emerged. The immune system′s inappropriate response was one of the main reasons for causing widespread damage in the body of a person infected with this virus [1]. Understanding the main pathways of pathogenesis to deal more efficiently with the damage caused by it became the goal of many studies [2]. Evidence suggests that SIRT serves as an upstream regulator of all the proposed pathways. SIRT1, a member of the sirtuin family, modulates diverse biological responses by controlling multiple target genes in cells. Following infection, SIRT1 orchestrates immune, inflammatory, autophagic, and metabolic responses, all required to establish and control host defenses [3]. Altogether, this study was aimed at investigating the possible role of SIRT1 in the pathogenesis of COVID‐19.

## 2. SIRT1: Structure and Function

SIRT1 is a Class III histone deacetylase (HDAC) that plays a significant role in regulating biological processes, including cellular senescence, apoptosis, metabolism, oxidative stress, inflammation, differentiation, and death, making it a potential therapeutic target in disorders and diseases. Even small changes in SIRT1 expression can significantly affect cellular responses. Silencing the SIRT1 gene increases inflammatory markers like TNF‐*α* and IL‐1*β*, underscoring its therapeutic potential for disease treatment [4]. SIRT1′s expression is regulated by factors like P53 and FOXO3a, which, respectively, inhibit and promote its transcription. Activation mechanisms of SIRT1 involve the NAD+/NADH ratio and posttranslational modifications [5]. Small molecules like resveratrol and taurine also enhance SIRT1 activity.

SIRT1 regulates responses during infections by modulating immune defenses and/or controlling inflammation. In sepsis, SIRT1 limits inflammation in myeloid cells, reducing organ failure and improving survival rates [6]. SIRT1 influences pathogen defense by regulating deacetylation of molecules like HMGB1. In *Salmonella*, SIRT1 and SIRT3 drive an immunometabolic switch in macrophages, affecting bacterial growth. SIRT1 also modulates antiviral responses by interacting with IFI16 and inhibiting its acetylation.

SIRT1 enables the FOXO family to regulate energy metabolism, programmed cell death, and cellular replication. The SIRT1/FOXO pathway influences oxidative stress, by boosting the expression of MnSOD and catalase [5]. Additionally, SIRT1 regulates autophagy and cell survival through pathways such as SIRT1‐FOXO1‐Rab7 and SIRT1‐AMPK, although the exact mechanism by which it promotes survival requires further clarification [3].

SIRT1 enhances tissue antioxidant capacity by activating PGC‐1*α* transcription and boosting the expression of enzymes like superoxide dismutase (SOD) and glutathione peroxidase (GPx). Additionally, SIRT1 deacetylates the nuclear factor kappa B (NF‐*κ*B) p65 subunit, affecting genes involved in inflammation (e.g., IL‐1, TNF‐*α*, IL‐8, and IL‐6) and apoptosis (e.g., IAPs, Bcl‐2, and TRAF1/2). SIRT1 also regulates lipid metabolism by deacetylating NF‐*κ*B [7]. Nrf2 activation promotes the expression of antioxidant proteins and regulates cellular redox balance while inhibiting inflammation. SIRT1 enhances Nrf2 stability and nuclear transport, improving cell resistance to oxidative damage [8].

## 3. Mechanisms of SIRT1 in Regulating Inflammation

SIRT1 regulates inflammation by deacetylating specific lysine residues on histones (e.g., H3K9) and key proteins (e.g., NF‐*κ*B), resulting in chromatin condensation and suppression of inflammation‐related genes, including IL‐6, TNF‐*α*, and IL‐1*β*. These actions on gene promoters are the major mechanisms by which SIRT1 exerts its anti‐inflammatory effects through direct inhibition of their transcription [9]. A key pathway involved in inflammation is the NF‐*κ*B signaling pathway, which stimulates glycolytic energy flux during acute inflammatory responses. Upon activation, NF‐*κ*B translocates to the nucleus, where SIRT1 can deacetylate its P65 subunit at lysine 310, suppressing the expression of inflammatory cytokines produced by NF‐*κ*B and preventing the spread of inflammation [10]. Furthermore, SIRT1 affects NF‐*κ*B′s DNA binding, effectively reducing its activity by activating AMPK, a key inhibitor of NF‐*κ*B. Conversely, NF‐*κ*B can regulate SIRT1 activity by upregulating interferon gamma (IFN*γ*) and reactive oxygen species (ROS) [11]. During inflammatory states, oxidative stress increases the production of ROS, which can oxidize cysteine residues in SIRT1, thereby inhibiting its activity and resulting in uncontrolled inflammatory cytokine production [12].

SIRT1 has been shown to reduce allergic airway inflammation by negatively regulating the mTOR and HIF‐1*α* (hypoxia‐inducible factor‐1*α*) signaling pathways. Both SIRT1 and HIF‐1*α* function as critical metabolic sensors in cellular metabolic processes, playing significant roles in immune responses. HIF‐1*α* synthesis is triggered by the activation of the PI3K/Akt/mTOR pathways. Under hypoxic conditions, HIF‐1*α* translocates to the nucleus, where it binds to hypoxia‐responsive elements (HREs) to upregulate various target genes, including glucose transporters (GLUTs), vascular endothelial growth factors (VEGFs), matrix metalloproteinases (MMPs), nitric oxide synthase (NOS), monocarboxylate transporters, hexokinase (HK), lactate dehydrogenase (LDH), and pyruvate kinase (PKM) [13]. HIF‐1*α* also participates in the secretion of cytokines and the cellular inflammatory response, highlighting its role in inflammation [14]. The SIRT1‐HIF‐1*α* axis connects innate immune signaling to adaptive immune responses by directing the production of inflammatory cytokines in dendritic cells independently of metabolism and promoting CD4+ T cell differentiation. Furthermore, the metabolism associated with SIRT1 and HIF‐1*α* inhibits mTOR activity and negatively regulates Th9 cell function. Given that immune cells are crucial for managing immune‐related diseases, the interplay between SIRT1 and HIF‐1*α* metabolism is closely linked to various immune‐related conditions, including infections, tumors, allergic airway inflammation, and autoimmune diseases [13].

Additionally, SIRT1 can also suppress inflammation through the activation of PPAR*α* and PGC‐1*α*, highlighting their multifaceted role in inflammatory regulation [15]. SIRT1 can inhibit P300‐mediated acetylation of the transcription factor activator protein 1 (AP‐1), which is responsible for enhancing the transcription of inflammatory cytokines including IL‐2 and IL‐8 [9] and downregulating cyclooxygenase‐2 (COX‐2) transcription, thereby exerting its anti‐inflammatory effects [16].

**Figure 1:**
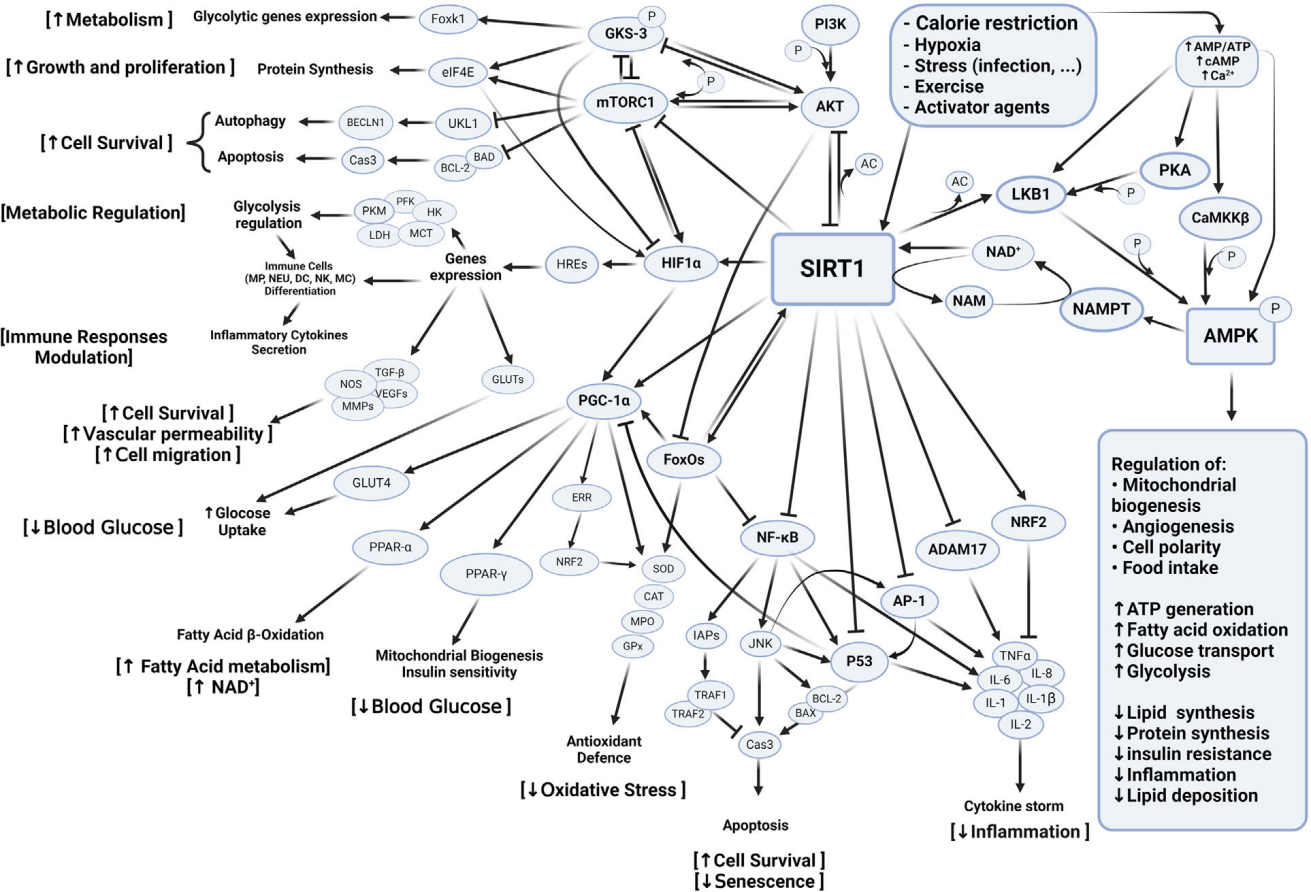
Mechanism of SIRT1 activation and function. SIRT1′s activity plays a crucial role in regulating gene expression, enabling the control of signaling pathways that impact various cellular functions including inflammation, oxidative stress, apoptosis, and mitochondrial activity. Various factors, including calorie restriction, hypoxia, stress, infection, and exercise, can play a role in the activation of SIRT1. Increased expression of S1 causes activation and inhibition of some metabolic pathways, by activating LKB1, HIF1, PGC‐1, FOXOs, and NRF2 which play an important role in metabolic regulation, cell survival, vascular permeability, blood sugar, and fatty acid metabolism. Also, SIRT1 plays a role in reducing inflammation and oxidative stress by inhibiting NF‐*κ*B, P53, and ADAM17 molecules. Abbreviations: SIRT1: silent information regulator 1, HIF1: hypoxia‐inducible factor 1‐alpha, PGC‐1: peroxisome proliferator–activated receptor gamma‐assisted activating factor‐1, FOXOs: forkhead box transcription factors, Nrf2: nuclear factor E2‐related factor 2, NF‐*κ*B: nuclear factor kappa B, NRFs: nuclear respiratory factor; ADAM17: a disintegrin and metalloprotease 17. This is an original figure that all parts of the figure, including all arrows and labels, were created by the authors at BioRender.com.

Factors such as aging, oxidative stress, hypertension, diabetes, and obesity can significantly deplete NAD+ levels, impairing SIRT1′s anti‐inflammatory functions. This depletion can lead to dysregulated inflammatory responses, lymphopenia, and other complications [5]. Excessive activation of PARP1 during inflammation can also lead to a decrease in SIRT1 activity by lowering the NAD+/NADH ratio [9]. Studies on COVID‐19 patients suggest that decreased NAD+ levels may limit SIRT1 activity, contributing to the excessive secretion of inflammatory cytokines and potentially triggering an excessive inflammatory response. This overactive immune response is associated with the acute phase of the disease and increased mortality rates among affected individuals [17]. Thus, SIRT1 emerges as a critical modulator of inflammation through multiple pathways, highlighting its potential therapeutic value (Figure 1).

## 4. COVID‐19 and SARS‐CoV‐2

SARS‐CoV‐2 is a highly pathogenic human coronavirus (CoV) first identified in Wuhan, China, in December 2019 [18]. While most individuals recover from COVID‐19, those with underlying health conditions are at greater risk for severe symptoms and mortality [19]. Cytokine storms, characterized by excessive inflammatory responses, play a crucial role in this, and their suppression has become vital for improving survival rates [20]. CoVs are known for their club‐shaped projections and have one of the largest RNA virus genomes, containing genes for vital proteins [21]. Human CoVs, including SARS‐CoV, MERS‐CoV, and SARS‐CoV‐2, may lead to severe pneumonia with mortality, while they can also cause mild cold‐like symptoms [22]. Studies indicate that excessive cytokine release, rather than the viral load, is more relevant to severe outcomes such as acute respiratory distress syndrome (ARDS), a leading cause of death in patients infected with SARS‐CoV and MERS‐CoV [23].

Research on COVID‐19 patients shows elevated levels of various cytokines, including IL‐1*β* and IFN‐*γ*, which activate T‐helper type 1 (Th1) responses essential for specific immunity. COVID‐19 patients also show increased T‐helper type 2 (Th2) cytokines like IL‐4 and IL‐10, which help mitigate inflammation [24]. Furthermore, levels of interleukin‐2 receptor (IL‐2R) and IL‐6 correlate positively with disease severity. This indicates a strong link between cytokine storm severity and overall disease severity [25]. In severe COVID‐19 cases, especially among elderly patients, mechanical ventilation and ARDS are common, which are characterized by damage to pulmonary and interstitial tissues caused by nonspecific inflammatory cell infiltration [26]. Excessive inflammatory response can also lead to multiple‐organ failure, explaining organ damage in some patients who do not experience respiratory failure [27].

## 5. The Role of SARS‐CoV‐2 Accessory Proteins

SARS‐CoV‐2 boasts a genomic architecture intricately composed of structural, nonstructural, and accessory proteins. The complex interplay between SARS‐CoV‐2 accessory proteins and host immune responses is akin to a delicate interplay that influences the course of infection. Findings underscore the role of accessory proteins, including ORF3a, ORF6, ORF7a, ORF7b, ORF8, and ORF10, in immune evasion.

Accessory proteins, such as ORF3a, ORF3b, ORF6, ORF7a, ORF7b, and ORF8, intricately participate in the orchestrated infection process of SARS‐CoV‐2. ORF6, ORF7a, ORF7b, and ORF8 act as interferon (IFN) antagonists, strategically hindering IFN signaling to ensure immune evasion, while ORF3a and ORF9b contribute to apoptosis and manipulate the host′s mitochondrial functions. Intriguingly, ORF3a emerges as a central factor, modulating host immune responses by activating proinflammatory cytokines, inducing NF‐*κ*B signaling, and influencing apoptosis and autophagy. The unique accessory protein, ORF8, distinguishes itself by interacting with host proteins, including major histocompatibility complex (MHC) class I molecules, resulting in their surface expression downregulation [28].

The diverse clinical spectrum of COVID‐19, ranging from mild to severe manifestations, is linked to the critical role of cytokine dysregulation and hyperinflammation, emphasizing the significance of understanding accessory protein functions. Despite significant progress, the roles of ORF7b and ORF10 remain enigmatic, warranting ongoing research to bridge existing gaps in our comprehension. The evolving understanding of these proteins offers potential targets for therapeutic interventions against COVID‐19, underscoring the need for continuing exploration [29]. In conclusion, the accessory proteins of SARS‐CoV‐2 form a complex molecular tapestry intricately woven into the fabric of viral pathogenesis [30].

## 6. SIRT1‐Related Signaling Pathways and COVID‐19

**Figure 2:**
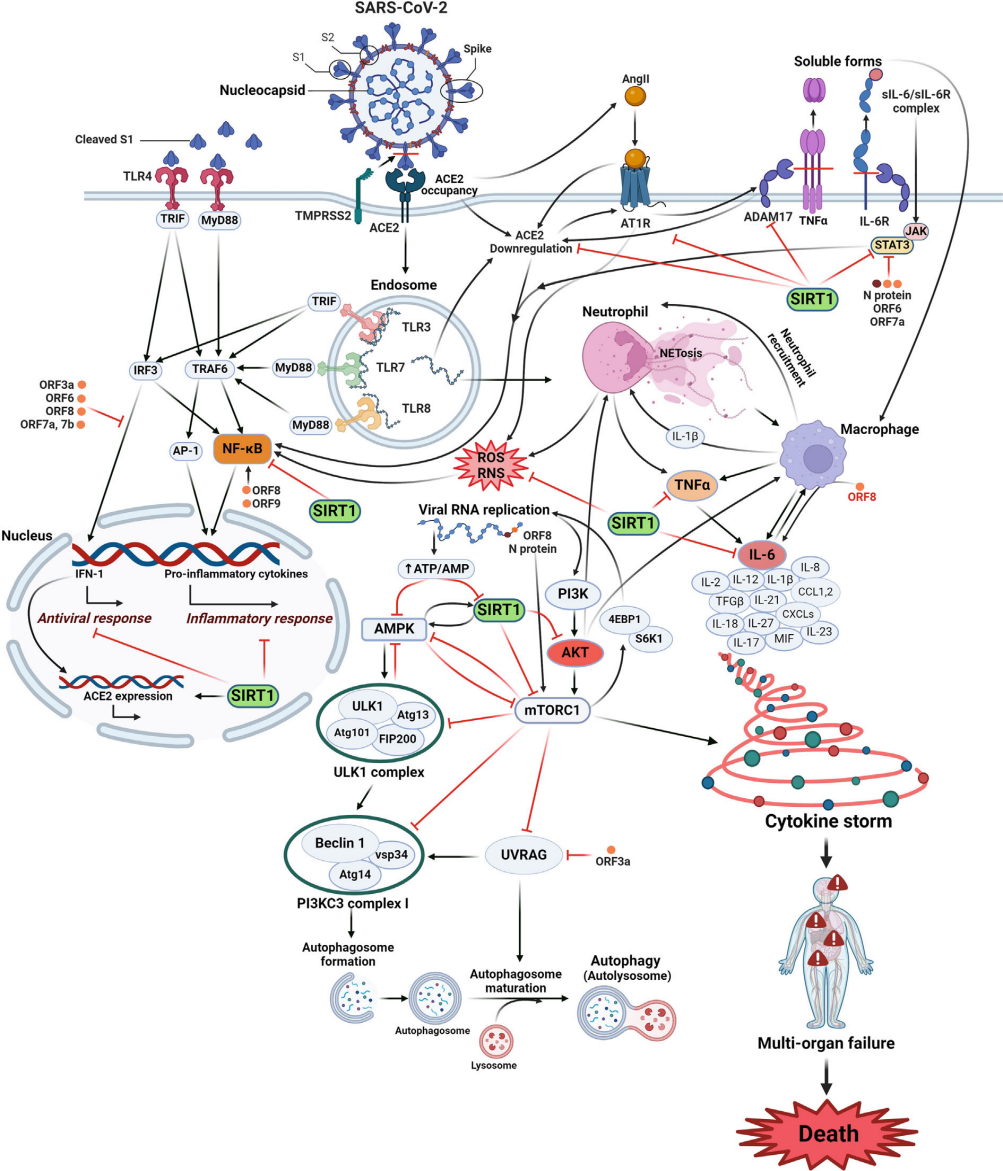
Signaling pathways upon SARS‐CoV‐2 infection and SIRT1 signaling pathways. This illustration highlights the critical signaling pathways activated during SARS‐CoV‐2 infection and the regulatory role of SIRT1. Upon viral entry, pathways such as NF‐*κ*B are activated, driving proinflammatory cytokine production and contributing to an overactive immune response. Simultaneously, oxidative stress and impaired autophagy exacerbate cellular damage. SIRT1 modulates these responses by suppressing NF‐*κ*B activity, reducing inflammation, promoting autophagy, and mitigating oxidative stress. Together, these pathways underscore SIRT1′s protective role in counteracting the detrimental effects of SARS‐CoV‐2 infection and suggest its potential as a therapeutic target in managing COVID‐19. This is an original figure that all parts of the figure, including all arrows and labels, were created by the authors at BioRender.com.

SIRT1 may act as an essential signaling hub in COVID‐19. SARS‐CoV‐2 entry could engage various signaling pathways that are intricately intertwined. SIRT1 may modulate each pathway to varying degrees, thereby influencing the inhibition and/or development of COVID‐19 in different ways [31]. In the following section, the signaling pathways activated in COVID‐19 and SIRT1′s possible role in their regulation are discussed (Figure 2).

## 7. ACE2 Interaction With SIRT1 in Virus Entry

The SARS‐CoV‐2 virus enters human cells by binding to the ACE2 receptor, which is expressed in various organs. The viral spike protein directly binds to ACE2, and the membrane serine protease TMPRSS2 cleaves the spike protein and the cytoplasmic tail of ACE2, facilitating viral entry. This process significantly increases in the presence of TMPRSS2 and decreases without it [32]. ACE2 is an enzyme that converts angiotensin II (Ang II) to Ang, which plays a protective role against the proinflammatory properties of Ang II. When ACE2 is occupied by the virus, the expression of Ang II increases, which subsequently increases soluble forms of IL‐6 receptor *α* (sIL‐6R*α*) and TNF*α* through a disintegrin and ADAM17, a metalloprotease enzyme, which plays a role in the activation of inflammatory cytokines [33, 34]. On the other hand, ACE2 surface expression is reduced after SARS‐CoV‐2 particles are internalized into endosomes. Thereby, reduced ACE2 anti‐inflammatory effect in turn aggravates proinflammatory cytokine secretion. Thus, ACE2 protection against heart failure, lung injury, and vascular permeability is impaired in COVID‐19 [35].

Along with the massive increase in inflammatory cytokines, downregulation of ACE2 and SIRT1 has been observed in COVID‐19 [36]. Studies have demonstrated that ACE2 expression could be upregulated at the transcriptional level by SIRT1 activity under energy stress conditions (e.g., hypoxia) [37, 38]; however, the pathophysiological consequences of this modulation are complex and context‐dependent. In early infection stages, increased ACE2 expression by SIRT1 may enhance SARS‐CoV‐2 viral entry, potentially elevating susceptibility to infection. Conversely, in later stages, ACE2 plays a protective role by maintaining endothelial and epithelial barrier integrity, reducing Ang II‐induced inflammation, vascular permeability, and subsequent organ damage. The protective effects of ACE2 vary among different organs. In the pulmonary system, ACE2 mitigates acute lung injury and attenuates the development of ARDS. ACE2 preserves myocardial function and reduces inflammatory damage in cardiac tissue. In the central nervous system, ACE2 may confer neurovascular protection, potentially limiting neuroinflammation. Therefore, SIRT1‐mediated ACE2 upregulation represents a dual‐edged mechanism, facilitating viral entry on one hand while preserving tissue homeostasis and function on the other. This duality highlights the necessity for precise therapeutic timing when modulating SIRT1 activity in COVID‐19 patients to balance antiviral defense and tissue protection [39–41].

## 8. Ang II/AT1R Axis and ADAM17

The attachment of the spike protein to ACE2 triggers Ang II binding to Ang II type 1 receptor (AT1R). AT1R activates several signaling cascades, such as NF‐*κ*B, generates ROS, and stimulates ADAM17, which increases inflammation by TNF‐*α* and IL‐6 receptor (IL‐6R) release [37]. On the other hand, overactivation of the Ang II/AT1R axis and ADAM17 following disease progression downregulates ACE2 surface expression, which in turn stimulates NF‐*κ*B and thus more cytokine secretion. All of these result in a vicious cycle of further uncontrolled hyperinflammatory response, which occurs in severe COVID‐19 [35, 42].

SIRT1′s role is most significant in the moderate to severe phase of COVID‐19, when the disease is progressing and the excessive inflammatory response is causing damage. SIRT1 activation could suppress AT1R expression both in vivo and in vitro [38], which may reduce COVID‐19‐mediated inflammatory response as well as death. Moreover, SIRT1 exerts anti‐inflammatory effects via ADAM17 downregulation, which is a TNF‐*α*‐converting enzyme [42, 43]. Increased TNF‐*α* levels inhibit ADAM17 activity in a negative feedback loop by SIRT1 stimulation, thereby inhibiting the formation of TNF‐*α* and subsequently affecting the production of IL‐1*β* and IL‐6, which are dependent on TNF‐*α*. An uncontrolled hyperinflammatory response may develop in COVID‐19 if ADAM17 suppression is not accrued by SIRT1 [44]. Thus, SIRT1 stimulation may improve the severity of the disease.

## 9. Toll‐Like Receptor (TLR) Axis

Innate immune cells are the first line of recognizing pathogens and rely on pattern recognition receptors (PRRs), termed pathogen‐associated molecular patterns (PAMPs). When these receptors (TLR3, TLR7, TLR8, and TLR4) bind to the viral RNA and spike protein, different signaling cascades proceed and then further generate proinflammatory cytokines, subsequently leading to a strong inflammatory response [34]. TLR3 activates the transcription factors interferon regulatory factor 3 (IRF3) via Toll/IL‐1R domain‐containing adaptor‐inducing IFN‐*β* (TRIF) to trigger transcription of type I IFNs (IFN‐1, e.g., IFN‐*α* and IFN‐*β*) or NF‐*κ*B. Thus, the TRIF‐dependent pathway induces antiviral and inflammatory responses [35]. TLR7 and TLR8 recruit myeloid differentiation primary response protein 88 (MYD88), which mediates the phosphorylation and activation of TNF receptor‐associated factor 6 (TRAF6). TRAF6 then causes the translocation of transcription factors (e.g., AP‐1 and NF‐*κ*B) into the nucleus, which in turn triggers transcription of various proinflammatory cytokines. Activated TLR4 recruits both TRIF‐ and MYD88‐dependent pathways to signal from its plasma membrane location [34]. Altogether, since TLRs activate NF‐*κ*B and IRF3, inducing IFN‐1 and proinflammatory cytokines during early to intermediate stages of SARS‐CoV‐2 infection [45], it seems that SIRT1 can help control inflammation and prevent progression to the acute phase and cytokine storm by inhibiting NF‐*κ*B and suppressing the immune response at these stages.

As the disease progresses, SARS‐CoV‐2 manipulates the host′s innate immune system by multiple mechanisms such as disrupting normal TLR signaling, leading to aberrant IFN‐1 responses and excessive inflammation, which exacerbate disease severity. Furthermore, the SARS‐CoV‐2–induced IFN‐1 responses also evidently increase cellular ACE2 levels, which may increase susceptibility to infection and inflammation [46]. To our knowledge, SIRT1 inhibits the gene expression of IFN‐1 and inflammatory cytokines at this stage by directly affecting transcription in the nucleus and prevents subsequent viral and inflammatory responses.

## 10. AMPK Pathways

The AMPK signaling pathway and its associated stimulatory and inhibitory processes play a role in the survival of viruses. Chawla et al. mentioned the mechanisms aiding in the SARS‐CoV‐2 evasion of host antiviral defense. SARS‐CoV‐2 hijacks the autophagy pathway at multiple steps. It should be noted that AMPK is the primary target by which SARS‐CoV‐2 affects the autophagic pathway. Although the exact mechanism is unknown, a high ATP/AMP ratio via SARS‐CoV‐2 active replication and release of the virus promoted the outpouring of ATP, which acts as a signaling molecule, reducing the phosphorylated, active form of AMPK (pAMPK). This process triggers a signaling cascade that affects AMPK downstream targets and exacerbates the inflammatory process [47, 48]. Mechanistically, by influencing the AMPK/ULK1/mTORC1 pathway, which, in turn, downregulates the ULK1 protein complex and increases mTORC1 activity, indeed, a triangular regulatory feedback loop exists among these three components [49]. The ULK1 complex (ULK1/Atg13/Atg101/FIP200) can activate the class III PI3 kinase complex 1 (PI3KC3) (Beclin 1/Atg14/vsp34). Activation of vsp34 recruits autophagy‐related proteins to facilitate phagophore formation and expansion. However, mTORC1 avoids autophagy triggering by ULK1 inhibition. Furthermore, SARS‐CoV‐2 can induce incomplete autophagy through the expression of ORF3a. Indeed, ORF3a interacts with the UV radiation resistance–associated gene (UVRAG), an autophagy regulator that promotes the fusion of phagosomes with lysosomes to form autolysosomes, and silencing UVRAG activity selectively suppresses PI3KC3‐C2 (Beclin1‐Vps34‐UVRAG) (ORF3a‐mediated incomplete autophagy facilitates severe acute respiratory syndrome coronavirus‐2 replication) [50]. In addition, ORF3a exhibits a strong interaction with VPS39, a facilitator of vesicle tethering, which disrupts the fusion of autophagosomes with the lysosome by blocking HOPS‐mediated SNARE complex assembly [51]. The SARS‐CoV‐2 viral nucleocapsid and ORF8 proteins can inhibit autophagy indirectly by inhibiting mTORC1 [52, 53]. As a result of these signals, the maturation of autophagosomes is blocked by hindering autophagosome–lysosome fusion. Gassen et al. confirmed the reduction of proteins responsible for phagophore formation and autophagosome–lysosome fusion in SARS‐CoV‐2–infected cells [54]. It has been stated that AMPK activation by metformin inhibits mTOR signaling and the NF‐*κ*B pathway, with subsequent IL‐6 and TNF‐*α* suppression and anti‐inflammatory IL‐10 activation. Moreover, metformin is a direct compound that activates SIRT1 [55]. Metformin, widely studied in diabetic and nondiabetic COVID‐19 patients, activates SIRT1 and AMPK pathways to reduce inflammation and enhance mitochondrial function, with systematic reviews and randomized controlled trials reporting decreased severity, hospitalization, and mortality rates, supporting its protective role in acute COVID‐19 [56, 57].

## 11. P53 Pathways

Studies have shown an increase in p53 expression in PBMCs of patients with COVID‐19 [58]. High levels of p53 expression were associated with a significant decrease in SIRT1 deacetylase expression and a higher expression of p21 [59]. The relationship between SIRT1 and p53 suggests that the deacetylation of p53 through SIRT1 can suppress p53 activity. In COVID‐19 patients, p53 and proinflammatory cytokine levels are increased, while SIRT1 expression in PBMCs decreases. The unbalanced p53/SIRT1 axis may impact lymphocyte homeostasis [60].

## 12. PI3K/Akt/mTOR Signaling

From a mechanistic perspective, AKT is a critical activator of mTORC1, and activation of the mTOR pathway leads to inhibition of autophagy. Most of the immune cells involved in the response to CoV are modulated by the PI3K/Akt pathway, and it seems that the PI3K‐*δ* isoform is responsible for modulating the function of CD8 T cells, Tregs, B cells, mast cells, and neutrophils. With the activation of PI3K/Akt, several cellular pathways and downstream proteins such as mTORC1, 4EBP1, and S6K1 are activated during viral infection, leading to the synthesis of viral proteins and increased cytokine secretion [61]. Inhibitors of the PI3K/Akt/mTOR pathway have been shown to exert antiviral effects, including specific anti‐SARS‐CoV‐2 effects. This indicates that targeting the PI3K/Akt pathway could represent a potential new weapon in the global fight against SARS‐CoV‐2 [62].

Activation of SIRT1 appears to be an intricate process, as we found that SIRT1 activates Akt only in the presence of growth factors. When nutrients are plentiful, SIRT1 promotes Akt signaling and cellular senescence. However, in the loss of insulin signaling, the lack of Akt‐mediated phosphorylation and SIRT1‐mediated deacetylation will facilitate the localization of FOXO into the nucleus, where it promotes the transcription of genes involved in endurance, stress resistance, and longevity, which suggests that SIRT1 may promote longevity under calorie‐restricted or growth factor–depleted conditions [63]. Akt is known to drive the macrophage inflammatory response. However, the mechanism by which Akt fine‐tunes the macrophage inflammatory response is poorly understood. Here, we found that Lys14 and Lys20 of Akt are deacetylated by SIRT1 during macrophage activation to suppress the macrophage inflammatory response. Mechanistically, SIRT1 promotes Akt deacetylation to inhibit the activation of NF‐*κ*B and proinflammatory cytokines. Loss of SIRT1 facilitates Akt acetylation and thus promotes inflammatory cytokine production in mouse macrophages, potentially worsening the progression of sepsis in mice. In contrast, the upregulation of SIRT1 in macrophages further contributes to the inhibition of proinflammatory cytokines via Akt activation in sepsis [64]. Akt has the most negative effects in the acute phase of COVID‐19. Therefore, targeting Akt at this stage and the recovery phase can modulate the repair and fibrosis process and contribute to clinical improvement. Taken together, targeted SIRT1 therapy should be performed with insight into the acute phase of the disease and with appropriate timing to be effective and safe [62].

## 13. NF‐*κ*B Pathway

In CoV infections, the NF‐*κ*B signaling pathway is activated through multiple signaling pathways. Virus replication in the host cell cytoplasm leads to the production and accumulation of double‐stranded RNA (dsRNA) (acting as a transcriptional mediator) in the context of the “canonical pathway” [65]. Upon binding dsRNA to a threonine kinase called PKR, NF‐*κ*B is activated [33]. Abnormal NF‐*κ*B activation is vital for the initiation and progression of multiple inflammatory respiratory diseases, including ARDS [66].

Additionally, NF‐*κ*B is activated through TLRs/TRIF‐ and/or MyD88‐dependent pathways [67–69], as described above. On the other hand, the Janus kinase/signal transduction and activator of transcription (JAK/STAT) pathway is required for the activation of the NF‐*κ*B pathway, with sIL‐6 binding to sIL‐6R as the main driver. When IL‐6 binds to its receptor, it activates the JAK‐STAT pathway; the phosphorylated STAT is translocated to the nucleus, resulting in an antiviral response with the induction of IFN‐1 and NF‐*κ*B activation [69, 70]. SARS‐CoV‐2 proteins (NSP5, ORF7a, N, and ORF6) affect JAK‐STAT signaling cascades by suppressing STAT phosphorylation and nuclear translocation, thus impairing host antiviral innate immunity independent of the level of IFNs [46]. Evidence suggests that the activation of the JAK/STAT pathway contributes to the subsequent worsening of COVID‐19 [45]. On the other hand, two nonstructural proteins of SARS‐CoV‐2, NSP13 and ORF9c, interact with NF‐*κ*B, suggesting that the virus can regulate the NF‐*κ*B signaling pathway [29]. Moreover, the overactivation of the Ang II/AT1R axis after ACE2 downregulation due to SARS‐CoV‐2 entry further induces NF‐*κ*B [71].

Another pathway leading to NF‐*κ*B activation is the mitogen‐activated protein kinase (MAPK) pathway, which in CoVs includes three p38 MAPK, Jun amino‐terminal kinases (JNK), and extracellular signal‐regulated kinases (ERK1/2), that are involved in viral pathogenesis [72, 73]. Ultimately, activated NF‐*κ*B can then translocate to the nucleus, where it binds to specific elements called *κ*B sites, initiating the transcription and production of proinflammatory mediators that further enhance NF‐*κ*B activation [67]. Therefore, this condition of hyperinflammation leads to a phenomenon called a cytokine storm, which worsens the patient′s condition and can even lead to death [69].

A significant focus has shifted toward developing JAK inhibitors as a therapeutic aid in COVID‐19 [45]. Pioglitazone, a PPAR agonist, has also been proposed as an effective treatment for COVID‐19 patients with Type 2 diabetes, cardiovascular complications, and hypertension. It works by reducing inflammatory parameters and inhibiting 3‐chymotrypsin‐like protease (3CLpro), thereby downregulating SARS‐CoV‐2 RNA synthesis and replication [74]. Evidence suggests that the PPAR*α*/*γ*–AMPK–SIRT1 pathway and fatty acid metabolism may be involved in influenza A virus (IAV) replication and its induced pneumonia [75]. These compounds work synergistically to inhibit NF‐*κ*B signaling and suppress inflammation [76]. In addition, SIRT1 acts as a primary defense against DNA and RNA viral pathogens. There is an inverse relationship between SIRT1 and virus replication; in fact, increased expression of SIRT1 directly reduces viral replication, thereby reducing the overactive immune response [77]. Therefore, SIRT1 stimulation might be a promising therapeutic strategy for SARS‐CoV‐2 infection.

## 14. MAPK Pathway

### 14.1. p38 MAPK Pathway

The p38 MAPK pathway may be disproportionately upregulated during SARS‐CoV‐2 infection due to ACE2 downregulation upon viral entry, as SARS‐CoV‐2 binds to and internalizes ACE2. Direct viral activation of p38 MAPK also promotes the lifecycle of respiratory viruses, including SARS‐CoV. Unrestrained p38 activation contributes to inflammation, thrombosis, and vasoconstriction, explaining the severe cardiac and pulmonary injuries seen in COVID‐19. Additionally, this pathway may facilitate viral entry through ACE2 endocytosis. p38 MAPK is a proinflammatory pathway linked to lung and heart injuries, and overactivation due to Ang II and viral proteins may further promote viral replication. The inhibitors of p38, currently in clinical development, show potential for COVID‐19 treatment [78].

### 14.2. Jun N‐Terminal Kinase (JNK) Pathway

The JNK signaling pathway, a crucial component of the MAPK superfamily, plays a significant role in regulating cell apoptosis, proliferation, and differentiation. Known as the “death pathway,” JNK is activated in response to various cellular stresses, including DNA damage and proinflammatory cytokines, which trigger apoptosis and inflammation while promoting the production of tissue cytokines. This pathway comprises two interconnected signaling routes: one that activates c‐Jun, Fos, P53, and apoptotic proteins such as BIM, BAD, and BAX, and another that suppresses cell survival pathways involving STATs and CREB. Research indicates that SARS‐CoV‐2 enhances the pathogenicity of the virus by activating both the JNK and JAK‐STAT pathways, resulting in increased cytokine levels, heightened inflammation, and a potential progression to pulmonary fibrosis. Notably, studies have demonstrated that SARS‐CoV infection in Vero E6 cells leads to JNK pathway activation, which prolongs disease duration [79]. Furthermore, JNK phosphorylation has been observed in cells infected with SARS‐CoV, as well as in those overexpressing various SARS‐CoV proteins. Despite these findings, the precise mechanisms of JNK activation during CoV infections and its involvement in virus‐induced apoptosis remain largely unexplored [35, 80].

### 14.3. Extracellular Signal–Regulated Kinases (ERK) Pathway

The ERK pathway, an integral part of the MAPK signaling cascade, exists in two isoforms: ERK1 (p44 MAPK) and ERK2 (p42 MAPK). This pathway is activated by extracellular ligands, facilitating the transmission of cellular signals to the nucleus. Research indicates that the spike and nucleocapsid proteins of SARS‐CoV‐2 activate the ERK pathway through the phosphorylation of ERK1/2. This activation subsequently induces the expression of proinflammatory factors such as COX‐2 and IL‐8 [80]. Furthermore, investigations into SARS‐CoV‐2 pathogenesis have demonstrated that pulmonary epithelial cells infected by the virus phosphorylate ACE2 through the action of casein kinase II (CK II), which leads to the activation of both the ERK1/2 and AP‐1 pathways. However, some studies have suggested that the inhibition of ERK1/2 does not affect the reduction of cells infected by SARS‐CoV‐2 [35].

## 15. Stage‐Specific Role of SIRT1 in COVID‐19 Progression

Emerging evidence suggests that the biological functions of SIRT1 in COVID‐19 pathogenesis vary significantly across different stages of the disease. Thus, targeted SIRT1 therapeutic interventions should be considered at the optimal timing according to the disease phase. During the initial phase of SARS‐CoV‐2 infection, SIRT1‐mediated upregulation of ACE2 may transiently increase cellular susceptibility to viral entry, while the effect of SIRT1 is not yet critical in this phase. In the acute or hyperinflammatory phase characterized by cytokine storm, SIRT1 exerts critical anti‐inflammatory effects by suppressing NF‐*κ*B activation and downstream proinflammatory cytokine secretion (e.g., IL‐6 and TNF‐*α*), thereby attenuating immune‐mediated tissue injury. This is the best time to intervene with SIRT1 activators to control inflammation, as well as enhance autophagy, potentially mitigating viral replication and dissemination.

Furthermore, in the postacute and recovery stages, SIRT1 contributes to cellular homeostasis by promoting mitochondrial function, reducing oxidative stress, increasing cellular tolerance to stress, helping to rebuild the immune system, and facilitating tissue repair mechanisms, which may reduce the risk of long‐term complications such as pulmonary fibrosis and neuroinflammation. Altogether, the phase‐specific functionality of SIRT1 in disease stages underscores the importance of optimizing the timing of SIRT1‐targeted therapies to maximize clinical benefits in COVID‐19 progression management [9, 42, 81, 82].

Evidence revealed that SIRT1 may play a crucial role in mitigating the persistent pathological features of long COVID‐19 through its multifaceted regulation of endothelial function, immune response, and oxidative stress pathways. Recent studies have demonstrated that long COVID‐19 is characterized by sustained endothelial dysfunction, including microvascular damage and impaired barrier integrity [83], processes that SIRT1 could be uniquely positioned to address, given its well‐documented protective effects on vascular tissue. Experimental models have shown that SIRT1 activation preserves endothelial function by enhancing nitric oxide bioavailability, reducing oxidative stress, and preventing vascular aging [84, 85], suggesting potential therapeutic applications for the vascular complications observed in post‐COVID‐19 syndrome.

Moreover, the persistent inflammatory state seen in long COVID‐19 patients may be modulated by SIRT1′s ability to suppress NF‐*κ*B signaling and promote regulatory T cell function [3, 86], while its capacity to counteract viral‐induced epigenetic modifications could reverse the long‐term immune reprogramming associated with SARS‐CoV‐2 infection [87]. The neurological manifestations of long COVID‐19, including brain fog and cognitive impairment as revealed by PET imaging studies [88], may also benefit from SIRT1 activation through its neuroprotective mechanisms involving microglial regulation and mitochondrial biogenesis [89]. Collectively, these findings position SIRT1 at the intersection of multiple pathological pathways in long COVID‐19, offering a promising molecular target for therapeutic intervention that could simultaneously address the vascular, immunological, and neurological sequelae of this complex condition.

## 16. Natural Activators of SIRT1

To combat the inflammatory cytokine storm, various therapeutic strategies have been proposed. Interferon lambda (IFN‐*λ*) shows potential by activating antiviral genes without overstimulating the immune response. Corticosteroids, despite concerns, can reduce mortality and hospital stays when used appropriately, although glucocorticoids should be used cautiously to prevent adverse effects [90] and other strategies. These approaches are aimed at modulating immune responses, suppressing inflammation, and restoring tissue homeostasis, ultimately improving patient outcomes in COVID‐19 treatment. While these strategies show promise, ongoing research is critical to determining their long‐term efficacy and safety in treating COVID‐19 patients. Natural compounds such as resveratrol and those listed in Table 1, all recognized as SIRT1 activators, have been extensively investigated for their therapeutic potential against COVID‐19 through a range of preclinical and clinical studies [118].

**Table 1:** Some of the most well‐researched natural stimulators of SIRT1.

**Compound**	**Signaling pathways involved**	**Effects**	**Effects on cytokines**	**Reference**
Resveratrol	– Activates SIRT1 to interrupt TLR4/NF‐*κ*B/STAT axis – Promotes SIRT1 binding to p65/RelA and NF‐*κ*B complex – Activates AMPK and controls SIRT1 activity via regulation of cellular levels of NAD+ – Promotes SIRT1 activity for deacetylation and activation of PGC‐1a	– Anti‐inflammatory – Anticancer – Antineurodegenerative – Immune response modulator – Cardiovascular protection – Energy sensor – Metabolic beneficial effects	– Activation of macrophages, T cells, and natural killer cells – Inhibits acetylation of RelA by reducing inflammatory factors such as TNF‐*α*, IL‐1*β*, IL‐6, metalloprotease (MMP)‐1, MMP‐3, and NF‐*κ*B‐mediated Cox‐2 – Suppression of CD4+ CD25+ regulatory T cells	[7, 91, 92]

Quercetin	– Upregulates SIRT1/AMPK axis to inhibit oxidative injury – Induces endothelial dysfunction – Increases SIRT1 expression and decreases NF‐*κ*B – Activates SIRT1/PGC‐1a and Bcl‐2/Bax pathways, exerting antiapoptotic effects	– Antioxidant – Anti‐inflammatory – Antiproliferative – Antiapoptotic – Chemoprevention – Anticarcinogenic – Immunostimulating – Reduce infection	– Induction of the expression of interferon‐g (IFN‐g) derived from Th‐1 and inhibition of IL‐4 derived from Th‐2 in normal peripheral blood mononuclear cells – Reducing T cell proliferation by blocking IL‐12‐induced tyrosine phosphorylation of JAK2, TYK2, STAT3, and STAT4 – Inhibiting COX and lipoxygenase	[7, 93–96]

Curcumin	– Inhibits prostaglandin synthesis, COX2, LOX, iNOS, cytokine production, and transcription factors – Eliminate ROS and RNS via induction of Nrf2/Keap1/ARE – Activates SIRT1 and reduces growth factor receptors, transcription factors, nitric oxide synthase, NF‐*κ*B, and TNFs – Increases NAD+ levels and AMPK activation	– Antioxidant – Anti‐inflammatory – Anticancer – Cardiovascular protection	– Reduce cytokine production –Suppress cytokine signaling (SOCS) and TLR4/MyD88/NF‐*κ*B axis	[97–100]

Hesperetin	– Increases SIRT1 expression through the AMPK/CREB pathway – Suppresses RelA/p65 acetylation to reduce NF‐*κ*B activity by inducing SIRT1 – Increase PGC‐1*α*, NRF1, and NFE2L2 via SIRT1 activation – Alleviates the SIRT1/NF*κ*B/NLRP3 pathway – Modulates the Sirt1/Nrf2 pathway	– Anti‐inflammatory – Antioxidant – Antiapoptotic – Cardiovascular protection – Neuroprotective – Antilipolytic – Hepatoprotective – Metabolic beneficial effects	– Reduce cytokine production via NF‐*κ*B suppression	[101–104]

Berberine	– Cell cycle regulation and promoting apoptosis – Apoptotic effect by inducing ROS production and increasing MAPK and JNK activity of p38 – Activates SIRT1 to modulate antioxidant and lipolytic effect of BBR in diabetes – Activating SIRT1/BBR decrease FOXO1 acetylation, triggering antiapoptotic signaling pathways via Bcl‐2 expression, and Bax and caspase‐3 downregulation	– Analgesic – Anticancer – Anti‐inflammatory – Myocardial protection – Antioxidant – Lipid metabolism	– Reduce cytokine production	[7, 105–108]

Fisetin	– Modulates ROS and immune response via AMPK/SIRT1 and Nfr2 pathways – Enhance SIRT1‐mediated PPAR and FOXO1 deacetylation – SIRT1/AMPKa reduction in lipid accumulation and an increase in lipolysis	– Anticancer – Cardiovascular protection – Anti‐inflammatory – Antioxidant – Neuroprotective – Metabolic beneficial effects	– Reduce cytokine production	[109–113]

Silibinin	– Abolishes oxidative stress – Inhibits PARP activation and restored NAD^+^ pool via activation of SIRT1/AMPK signaling – Regulated lipid metabolism via SIRT‐1/PPAR*α* activation – Increases PGC‐1*α* and UCP gene expression	– Antioxidant – Anti‐inflammatory – Anticancer – Antimicrobial – Antidiabetic – Hepatoprotective – Antiarthritis	—	[113–115]

Luteolin	– Upregulates SIRT1/FOXO1/PGC1 activity to inhibit ROS production – SIRT1/Nrf2/TNF‐*α* pathway to be critical for the hepatoprotective effect of luteolin – Regulates Keap1/Nrf2/ARE signaling, inhibited inflammation, and prevented apoptosis via regulation of Bax, p53, and Bcl‐2 expression mediated by SIRT1 activation	– Antioxidant – Anti‐inflammatory – Anticancer – Antimicrobial – Chemoprevention	– Suppressed inflammatory cytokine release via NF‐*κ*B suppression and p38 MAPK	[113, 116, 117]

Resveratrol has shown antiviral and anti‐inflammatory effects by inhibiting SARS‐CoV‐2 replication and modulating inflammatory pathways, with clinical trials indicating reduced hospitalization and improved outcomes in mild COVID‐19 patients, though larger studies are needed for confirmation [119–124]. Quercetin has demonstrated promising results in randomized clinical trials by reducing hospitalization duration, oxygen therapy requirements, and disease progression, while accelerating viral clearance and symptom resolution [125, 126]. Curcumin exhibits antiviral activity through inhibition of viral replication and modulation of key inflammatory pathways such as NF‐*κ*B, supported by in vitro and computational studies, although direct in vivo evidence remains limited, necessitating further research [127, 128]. Hesperidin has been evaluated in randomized controlled trials showing modest symptom improvement and reductions in inflammatory markers, but findings lack statistical significance, highlighting the need for larger trials [129, 130].

Berberine has shown antiviral and anti‐inflammatory effects in preclinical studies against SARS‐CoV‐2. However, a pilot clinical trial in COVID‐19 outpatients found no significant benefit of berberine on symptom improvement or inflammatory markers. These results indicate that more rigorous and larger clinical studies are needed to confirm berberine′s therapeutic potential and optimal dosing in COVID‐19 treatment. Thus, current clinical evidence is insufficient to fully support its efficacy in patients [131]. Fisetin has shown promising results in preclinical SARS‐CoV‐2 models by reducing inflammation and mortality. It is currently being evaluated in Phase 2 clinical trials targeting older adults and high‐risk COVID‐19 patients to assess its efficacy in preventing disease progression and complications. Advances in fisetin formulations have improved its bioavailability, supporting its clinical use. While definitive clinical outcomes are pending, fisetin represents a promising adjunctive therapy for COVID‐19 [132, 133].

Silibinin offers a dual mechanism by targeting viral replication and the cytokine storm, with preclinical data supporting its antiviral and anti‐inflammatory effects; clinical trials are underway to assess its safety and efficacy in moderate to severe COVID‐19 patients, though more extensive in vivo research is required [134]. Collectively, these natural SIRT1 activators hold significant promise as adjunctive therapies in COVID‐19, yet comprehensive in vivo studies and large‐scale, well‐designed clinical trials remain essential to establish their efficacy, optimal dosing, and mechanisms of action in the context of SARS‐CoV‐2 infection [135]. Additionally, some of these compounds demonstrate anticancer, neuroprotective, and metabolic benefits. Collectively, these natural compounds, as SIRT1 activators, hold great potential for use in treating diseases associated with inflammation and oxidative stress, with detailed signaling pathways involved in these effects presented in Table 1.

## 17. Conclusion

SIRT1, a NAD+‐dependent deacetylase enzyme, plays a pivotal role in regulating numerous cellular and physiological processes. Recent studies have highlighted its significant impact on the body′s defense mechanisms against SARS‐CoV‐2 infection. One of SIRT1′s key attributes is its ability to modulate inflammatory responses through pathways involving NF‐*κ*B. As a central factor in inflammation regulation, NF‐*κ*B becomes hyperactive in viral infections, including COVID‐19, leading to severe inflammatory states and cytokine storms. SIRT1 activation can suppress NF‐*κ*B activity and reduce the secretion of proinflammatory cytokines such as IL‐6 and TNF‐*α*. This mechanism is particularly vital for patients with severe COVID‐19, who are prone to excessive inflammatory responses. Beyond inflammation control, SIRT1 plays a critical role in enhancing cellular metabolism, reducing oxidative stress, and promoting autophagy. Oxidative stress, driven by excessive free radicals and reduced antioxidant capacity, is a significant contributor to cellular damage during COVID‐19 infection. By increasing SIRT1 activity, cells can better combat oxidative stress, thereby minimizing its detrimental effects. Moreover, SIRT1‐driven autophagy facilitates the clearance of viral particles and reduces viral replication. Therefore, enhancing SIRT1 activity through pharmacological or dietary interventions, such as resveratrol or SIRT1 activators, has emerged as a potential therapeutic strategy. In summary, SIRT1 plays a protective role against COVID‐19 by precisely regulating inflammation, oxidative stress, autophagy, and immune balance. Further research into this area could pave the way for more effective therapeutic approaches to alleviate the disease burden and improve outcomes for COVID‐19 patients.

## Disclosure

All authors reviewed the manuscript. All authors agree to be accountable for the research presented.

## Conflicts of Interest

The authors declare no conflicts of interest.

## Author Contributions

H.S‐A., H.M., P.K., F.S.J., Z.B., E.Y.A., D.I., and Y.M. wrote the main manuscript text. H.S‐A. prepared Figures 1 and 2.

## Funding

This research did not receive any specific grant from funding agencies in the public, commercial, or not‐for‐profit sectors.

## Data Availability

The data that support the findings of this study are available on request from the corresponding authors. The data are not publicly available due to governmental policy and privacy.
